# NRN-EZ: an application to streamline biophysical modeling of synaptic integration using NEURON

**DOI:** 10.1038/s41598-022-27302-8

**Published:** 2023-01-10

**Authors:** Evan A. W. Cobb, Maurice A. Petroccione, Annalisa Scimemi

**Affiliations:** 1 Department of Biology, SUNY Albany, 1400 Washington Avenue, Albany, NY 12222-0100 USA; 2 Department of Computer Science, SUNY Albany, 1400 Washington Avenue, Albany, NY 12222-0100 USA

**Keywords:** Neural circuits, Neuroscience, Mathematics and computing

## Abstract

One of the fundamental goals in neuroscience is to determine how the brain processes information and ultimately controls the execution of complex behaviors. Over the past four decades, there has been a steady growth in our knowledge of the morphological and functional diversity of neurons, the building blocks of the brain. These cells clearly differ not only for their anatomy and ion channel distribution, but also for the type, strength, location, and temporal pattern of activity of the many synaptic inputs they receive. Compartmental modeling programs like NEURON have become widely used in the neuroscience community to address a broad range of research questions, including how neurons integrate synaptic inputs and propagate information through complex neural networks. One of the main strengths of NEURON is its ability to incorporate user-defined information about the realistic morphology and biophysical properties of different cell types. Although the graphical user interface of the program can be used to run initial exploratory simulations, introducing a stochastic representation of synaptic weights, locations and activation times typically requires users to develop their own codes, a task that can be overwhelming for some beginner users. Here we describe NRN-EZ, an interactive application that allows users to specify complex patterns of synaptic input activity that can be integrated as part of NEURON simulations. Through its graphical user interface, NRN-EZ aims to ease the learning curve to run computational models in NEURON, for users that do not necessarily have a computer science background.

## Introduction

Information transfer within the brain relies on the ability of individual neurons to integrate synaptic inputs in space and time. In most instances, synaptic integration behaves as a non-linear system, where neither analytical solutions nor intuition can guide our understanding of the operating principles of neurons and circuits. To overcome this limitation, back in the ‘80 s, scientists began to develop approaches to efficiently compute the Hodgkin Huxley branched cable equations^1–3^. These works laid the foundation for the development of realistic quantitative models through which investigators could determine how non-homogeneous ion channel distribution in neurons with complex architectures controls synaptic integration and action potential firing. The software NEURON was developed exactly for this purpose in the mid-1980s^1,4–6^. Over the course of years and decades, NEURON has gone through extensive upgrades in features and performances. To this day, NEURON remains one of the most widely used simulation environments for biologically detailed biophysical simulations, with a vibrant user community, readable documentation, and an extensive online ModelDB database^7–13^. Its use continues to be instrumental to cross-validate experimental data, estimate experimentally inaccessible parameters and establish relationships between specific patterns of synaptic activity and firing output profiles. Therefore, de facto, NEURON has become a standard and up-to-date tool that the neuroscience community continues to use for single-cell or network simulations^14^.

In its original release, NEURON relied on an interpreted programming language based on a floating-point calculator with C-like syntax, called hoc (an acronym for high order calculator)^15^. Through hoc codes, users defined specific parameters in the simulations, analyzed data, calculated new variables, etc. An improved hoc interpreter was developed in 1985 by the Computing Science Research Center at Bell Labs but was not generally adopted by commercial Unix systems or Linux distributions, which instead relied on developments of earlier calculator languages like dc and bc. For historical reasons, NEURON continued to rely on hoc and hoc interpreters for some time, whereas ion channel mechanisms (e.g., Hodgkin-Huxley-like kinetics, Markov kinetics, synaptic properties, etc.) were programmed using templates written as MOD files^13,16^. These templates were then translated into functions in C, compiled and assembled into a dynamic library^12^.

In 2006, the NEURON simulation environment was extended to support parallel processing^17^, but some potential drawbacks, including the lack of a debugger and profiler, and some of the intrinsic limitations of the hoc language, remained^12^. Other tools, like neuroConstruct, based on the use of new simulator-independent NeuroML standards, were created to allow importing complex cell morphologies to be used and implemented for single cell or network models in NEURON and other simulators^18^. As the use of Python became even more popular in the neuroscience community, it became possible to describe individual neurons and networks with NEURON not only using the native hoc language, but also using Python or a mix of Python and hoc using PyNEURON^19^. Among other advantages, Python offered a larger choice of data structures for data organization, communication, compatibility and portability with other simulator and analysis packages that use Python as their interpreter language (e.g., NEST, MOOSE, Auryn, GENESIS, PyNN, BRIAN)^20–30^. At the time, this implementation allowed users to run simulations of moderate size neural networks (i.e., composed of 1∙10^4^–1∙10^6^ cells)^20^. Newer versions of hoc continued to be implemented as part of the Plan 9 operating system by Bell labs in 2015, and considerable extensions continued to be made after that.

Automation of many network modeling requirements was aided by the development of NetPyNE, an open-source Python package that serves as a high-level declarative NEURON wrapper^31^. NetPyNE allowed building large-scale networks and running parallel simulations, ensuring replicability, parameter optimization and analysis, which until this point were left to be implemented by each user^31–33^. Together with neuroConstruct and other tools that became available over time (e.g., PyNN, Topographica, ARACHNE and BioNet^22,34–36^) NetPyNE streamlined network modeling with NEURON. Since its release, it has been integrated with other tools available in the neuroscience community including the Human Neocortical Neurosolver, the Neuroscience Gateway Portal, and the Open Source Brain and EBRAINS platforms^37–44^.

In the more recent age of big data and hardware developments, NEURON embraced the portability challenge (i.e., running on different operating systems and making good use of multi-core CPUs and GPUs), while also being able to run jobs quickly, accurately, and with backward compatibility. This was accomplished with a recent modernization and modularization of NEURON. Briefly, by integrating NEURON/NetPyNE with CoreNEURON (a scalable simulation engine) and NMODL (a compiler capable of targeting CPUs and GPUs), users can now run multi-scale models and reaction–diffusion simulations using a software that offers improved sustainability, portability, and performance, and that can run on either hoc or Python^14^.

While these modeling tools grow in complexity and accessibility, now more than ever computational proficiency and literacy have become essential skills in a biologist’s toolbox, guiding data interpretation and experiment design. Despite all the improvements, hoc has not completely gone off the shelf, nor are some potential limitations inherent not only to hoc but also to the Python implementations of NEURON. For example, at present, the spatial distribution and activation time of different synaptic inputs can be hard coded by users or arranged based on the identity of different sub-cellular compartments but not, say, based on the distance from a given reference point from the soma. Also, it may be useful to run published models using hoc, without converting them to Python. From an educational perspective, it is important to ease the learning curve for trainees to run simulations at a single cell level, before scaling them up to the level of neuronal networks. Although learning how to code may not be an insurmountable task for a computer scientist, it can still be intimidating for many biologists that have not received formal or informal training in programming. Therefore, while we integrate programming as a core skill for biologists, it is also important to make all the tools necessary to run biophysical models of neurons more accessible to the wider scientific community and junior trainees.

To overcome these hurdles for generating models, we developed an open-source application called NRN-EZ, which is accessible to users with limited programming background. NRN-EZ allows users to: (i) load the 3D morphology of a digitally reconstructed neuron; (ii) add user-defined excitatory and inhibitory synaptic inputs; (iii) generate uniform or Poisson distributions for synaptic location, and activation time of different types of synaptic inputs, or any user-defined distribution for synaptic weight; and (iv) automatically generate .hoc and .py files that can then be compiled in NEURON, providing users the option to run simulations in NEURON using either the hoc or Python interface. The NRN-EZ code is freely accessible through our Github repository, it is easy to use and can be modified by users as they see fit.

## Results

### Overview of the graphical user interface

**Figure 1:**
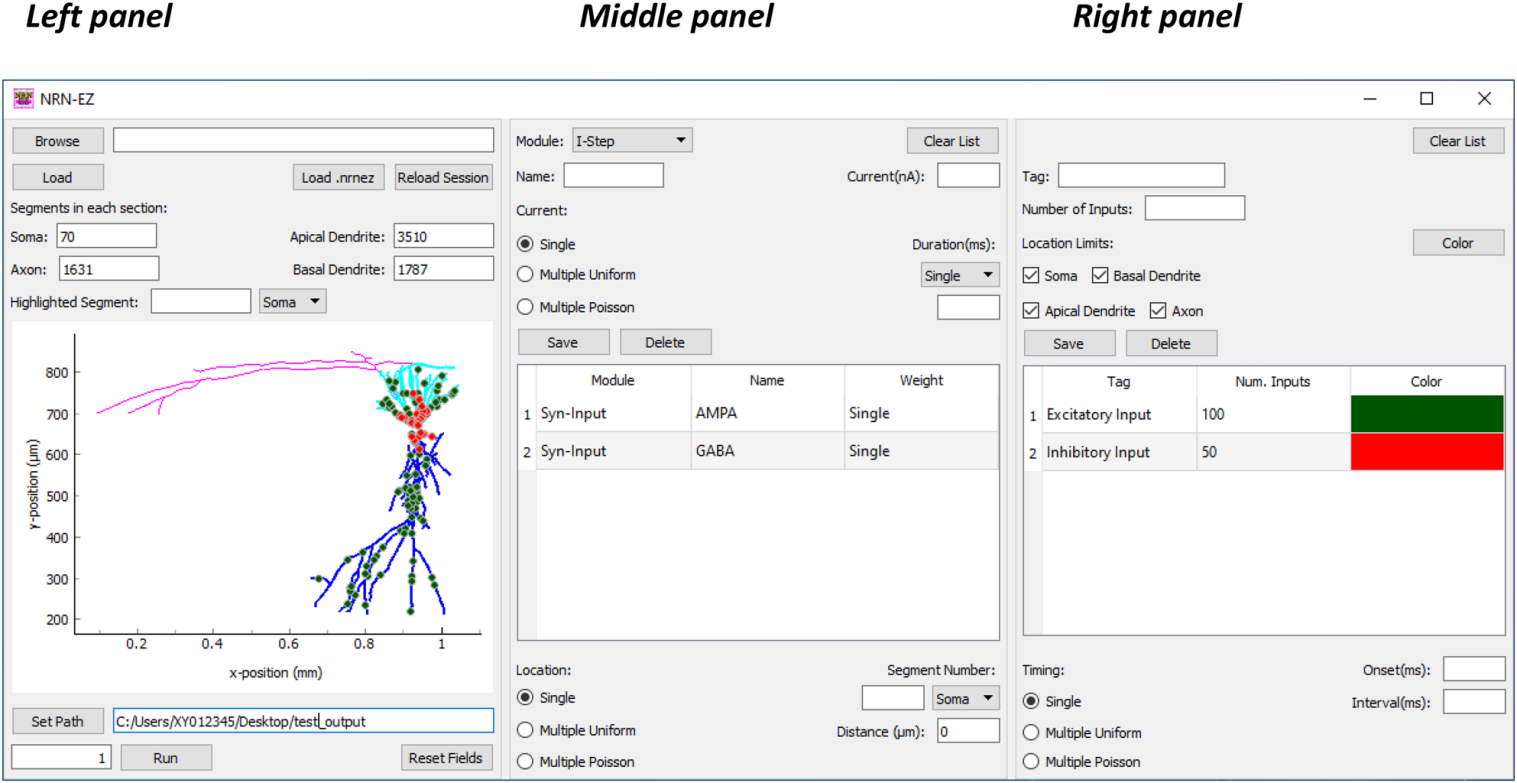
The NRN-EZ GUI. The GUI is composed of a left, middle, and right panel. The left panel allows users to upload a file containing information about the 3D neuron structure. Once the upload is finished, the users can see the number of segments in each of the four sections of the neuron. They can also pan through the neuron morphology, identify specific segments, and set the path to the directory, which will contain the output from NRN-EZ. The middle panel is the one through which users specify the type of module they plan to use: current steps or synaptic stimulations. They can use multiple modules and assign them an arbitrary name. The weight, kinetics and timing of activation can be selected through the parameters described in the upper portion of the panel. The bottom portion allows users to select the location and spatial distribution of these inputs. The right panel allows selecting the number of inputs and the limits for their spatial location. For example, one may decide to confine synaptic inputs of a given type only to one dendritic compartment. NRN-EZ allows selecting multiple types of inputs, with unique locations and weights that can be integrated to run NEURON simulations of synaptic integration. The neuron morphology loaded in the GUI has color-coded sections and synaptic inputs.

**Figure 2:**
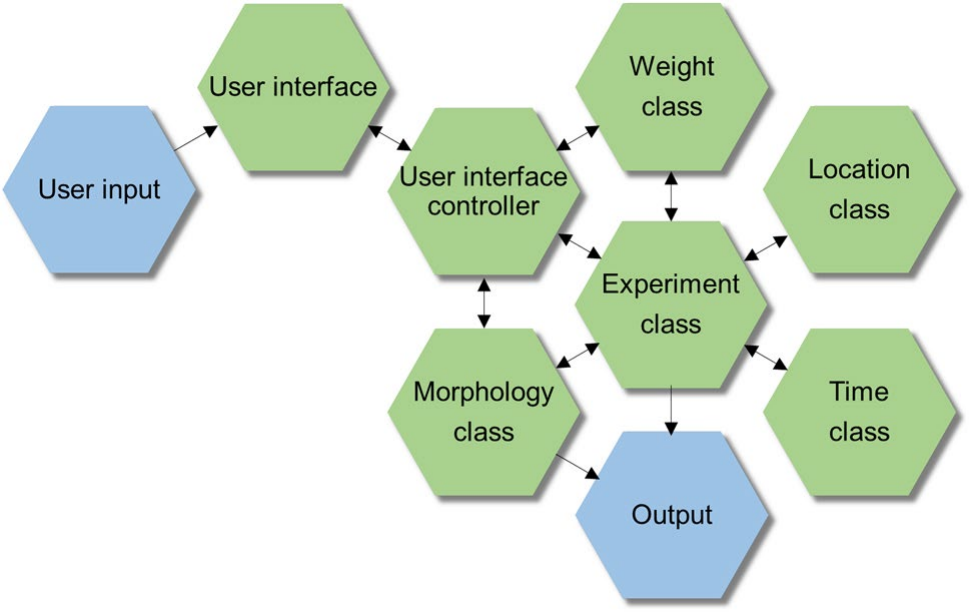
Software architecture of NRN-EZ. Schematic representation of the fundamental structure and design for the NRN-EZ software. The blue hexagons highlight the start and end points of the logical workflow (i.e., the parameter choice from the GUI and the output file generated using NRN-EZ. The green hexagons identify intermediate workflow steps.

**Figure 3:**
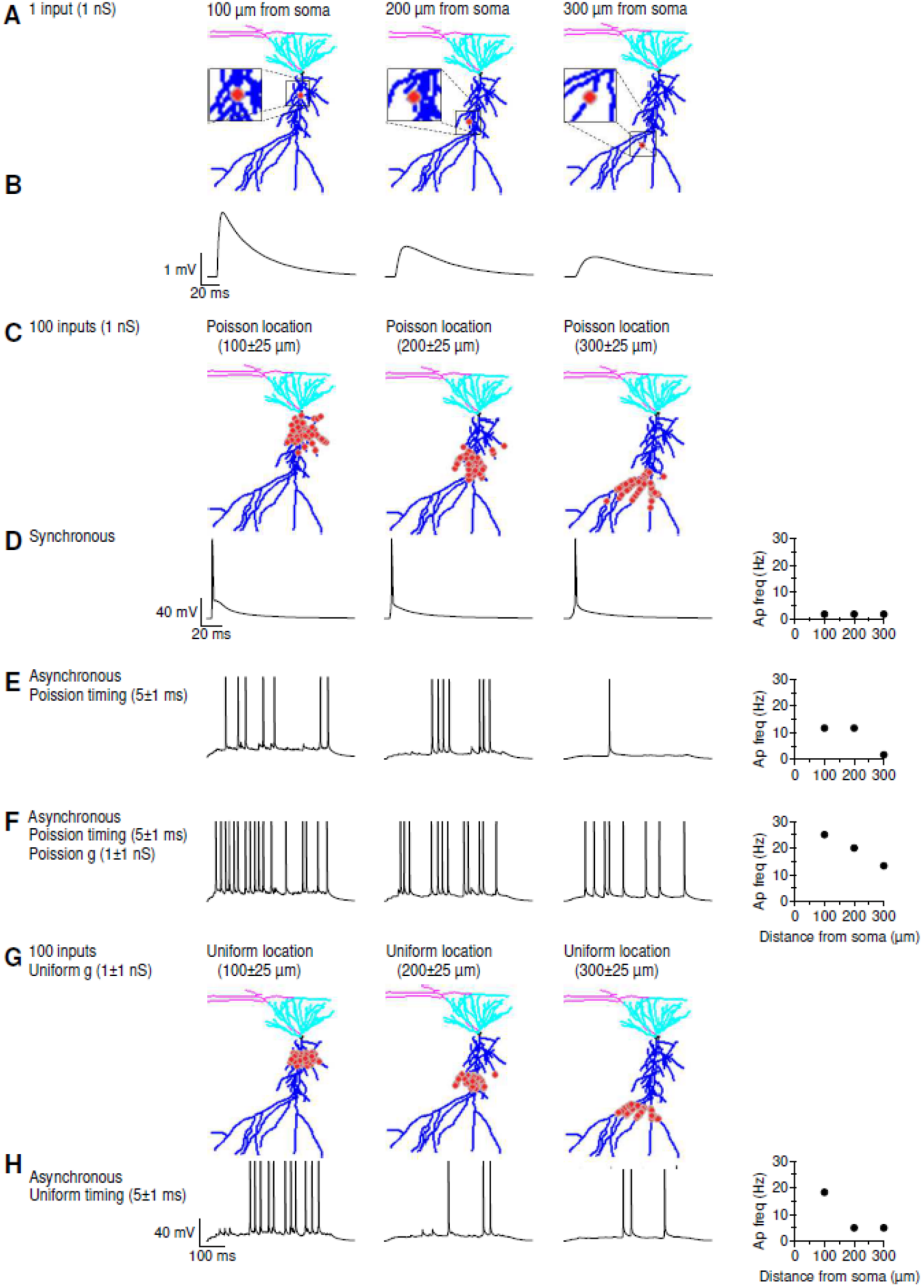
Sample Application of NRN-EZ for summation of supra-threshold EPSPs. (**A**) We used NRN-EZ to place one AMPA excitatory input at 100–300 µm from the soma of a 3D reconstructed, biocytin-filled CA1-PCs. The maximum intensity projection of the CA1-PC includes the soma (*black*), basal dendrites (*cyan*), apical dendrites (*blue*) and the location of the input (*red*). The synaptic weight was set to 1 nS. (**B**) Somatic recording obtained using the NEURON simulation environment. The action potential frequency recorded at the soma for inputs located at increasing distances is plotted in the right panel. (**C**) As in A, for 100 inputs with a Poisson placement along the distal dendrite of the CA1-PC. (**D**) As in B, in response to the synchronous activation of the 100 inputs described in C. (**E**) Somatic membrane potential recorded in response to the asynchronous activation of the 100 excitatory inputs described in C. (**F**) As in E, for synaptic inputs with a Poisson conductance distribution. (**G**) As in C, for 100 inputs with a uniform spatial distribution centered 100–300 µm away from the soma. (**H**) As in F, for 100 inputs with uniform spatial distribution and uniform timing of activation.

**Figure 4:**
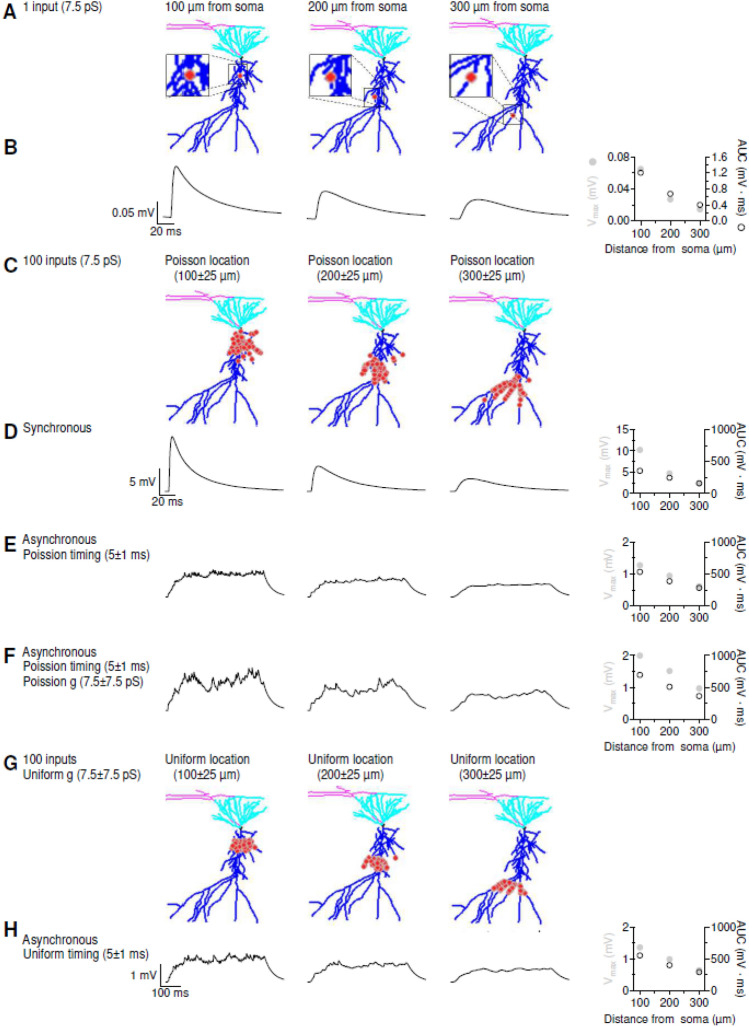
Sample Application of NRN-EZ for summation of sub-threshold EPSPs. (**A**) We used NRN-EZ to place one AMPA excitatory input at 100–300 µm from the soma of a 3D reconstructed, biocytin-filled CA1-PCs. The maximum intensity projection of the CA1-PC includes the soma (*black*), basal dendrites (*cyan*), apical dendrites (*blue*) and the location of the input (*red*). The synaptic weight was set to 7.5 pS. (**B**) Somatic recording obtained using the NEURON simulation environment. The maximum depolarization (V_max_) and the AUC of the somatic EPSP for increasing distances from the soma are plotted in the right panel. (**C**) As in A, for 100 inputs with a Poisson placement along the distal dendrite of the CA1-PC. (**D**) As in B, in response to the synchronous activation of the 100 inputs described in C. (**E**) Somatic EPSPs recorded in response to the asynchronous activation of the 100 excitatory inputs described in C. (**F**) As in E, for synaptic inputs with a Poisson conductance distribution. (**G**) As in C, for 100 inputs with a uniform spatial distribution centered 100–300 µm away from the soma. (**H**) As in F, for 100 inputs with uniform spatial distribution and uniform timing of activation.

**Figure 5:**
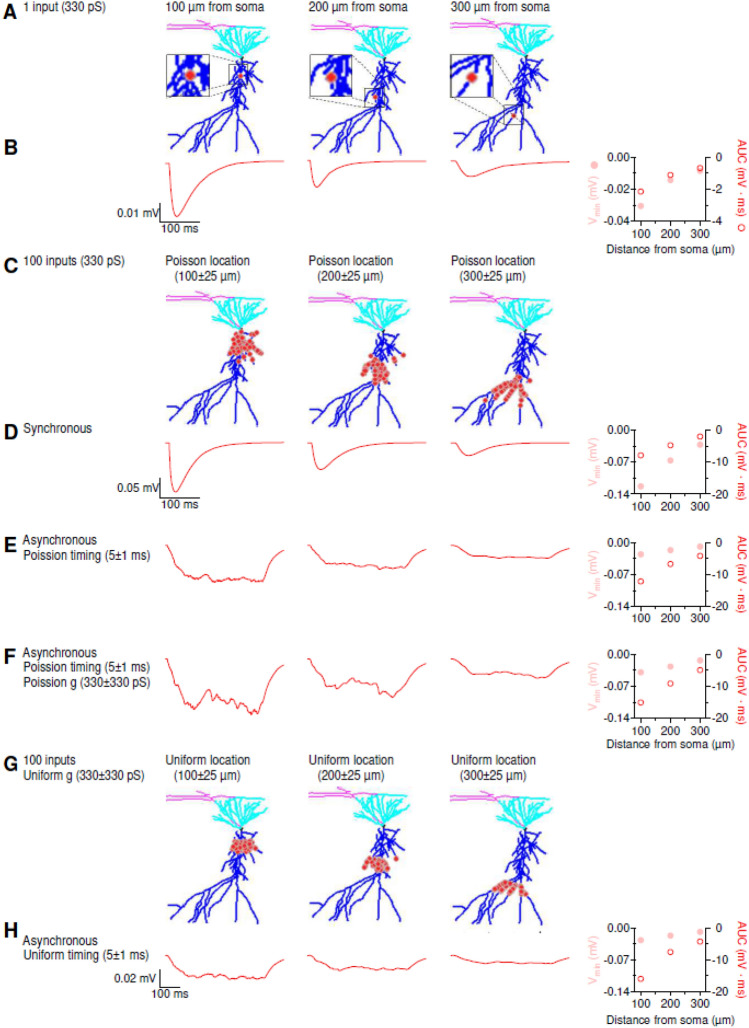
Sample Application of NRN-EZ for summation of IPSPs. (**A**) We used NRN-EZ to place one GABA_A_ inhibitory input at 100–300 µm from the soma of a 3D reconstructed, biocytin-filled CA1-PCs. The maximum intensity projection of the CA1-PC includes the soma (*black*), basal dendrites (*cyan*), apical dendrites (*blue*) and the location of the input (*red*). The synaptic weight was set to 330 pS. (**B**) Somatic recording obtained using the NEURON simulation environment. The minimum value of the membrane potential (V_min_) and the AUC of the somatic IPSP for increasing distances from the soma are plotted in the right panel. (**C**) As in A, for 100 inputs with a Poisson placement along the distal dendrite of the CA1-PC. (**D**) As in B, in response to the synchronous activation of the 100 inputs described in C. (**E**) Somatic IPSPs recorded in response to the asynchronous activation of the 100 inhibitory inputs described in C. (**F**) As in E, for synaptic inputs with a Poisson conductance distribution. (**G**) As in C, for 100 inputs with a uniform spatial distribution centered 100–300 µm away from the soma. (**H**) As in F, for 100 inputs with uniform spatial distribution and uniform timing of activation.

NRN-EZ has a simple and intuitive graphical user interface (GUI), organized in three adjacent panels (Fig. 1), and a streamlined software architecture (Fig. 2). Through the GUI, users can load the 3D structure of a biocytin-filled and reconstructed cell (which is displayed as a 2D maximum intensity projection in the NRN-EZ interface) and populate it with inputs of varying weights, spatial distribution, and temporal patterns of activation. The 3D morphology of a cell can be generated de novo from 3D reconstructions of biocytin-fills using open source or proprietary software (e.g., SimpleNeuriteTracer, Bitplane Imaris, etc.). Alternatively, one can use 3D reconstructions available from publicly accessible repositories like NeuroMorpho.org (https://www.neuromorpho.org)^45,46^. Each reconstruction is typically stored as a .swc file, an open-source file format widely used in the neuroscience community. Other file formats can be converted to .swc by using simple and freely available command-line utilities. In each .swc file, cells are defined through a list of sub-cellular compartments, which users can populate with arbitrary passive and active conductances. A typical neuron contains four types of compartments: the soma, the axon, the apical and the basal dendrites. Each compartment is identified by its 3D Cartesian coordinates, its radius and parent compartment. The .hoc or .py files generated through NRN-EZ can then be imported in NEURON to analyze, for example, the spiking output of a neuron (see examples in Figs. 3, 4 and 5).

### The left panel of the GUI: visualizing cells and setting the path for the working environment

The first step when using NRN-EZ is to either: (i) load (i.e., import) a .swc file, (ii) reload a .pkl file, which allows users to recreate the morphology and reload all input parameters of a cell (e.g., synaptic weight, onset, etc.); or (iii) load a .nrnez file, which allows users to load some default values into the GUI, including values for different input parameters. The .swc file can be loaded by first navigating to its location folder using the “Browse” button in the top left corner of the GUI, and by clicking the “Load” button below it. All these files (i.e., .swc, .pkl and .nrnez) are opened using the Python open function (with the Pkl and JSON library used to read .pkl and .nrnez files, respectively). NRN-EZ only imports .swc files and does not correct for potential inaccuracies and discontinuities in the morphology reconstructions, which can still be done using NEURON’s Import3D tool. It also does not have a built-in CellBuilder, which can still be used directly from NEURON. The .swc file format is widely used and standardized to store neuron morphologies and share digitally reconstructed neurons. There are simple command-line utilities freely available for converting other 3D neuron morphology formats into .swc (e.g., MorphoML, Genesis, Imaris, NeuroZoom, Eutectics, etc.), like NeuronLand (http://www.neuronland.org). In our custom load of .swc files, each line of the file is read, and the data is saved into an “nPart” object, which describes a single segment of a cell morphology. During loading, we count the total number of segments of a given type (i.e., soma, axon, apical, basal). A message box is displayed to the user if segments without an assigned segment type are detected. The nPart object holds the segment id, type, *xyz* position, radius, and parent id of the segment. After the synaptic inputs have been generated, the experimental settings have been defined and the file generation occurs, then a .nrn file with the morphology definition is created using the .swc file. This .nrn file will be loaded in NEURON. Since .swc files define segments by points and the .nrn file defines segments by lines, the values in the .swc are used to calculate the segment definitions needed in the .nrn file. For example, given soma id *a* with parent id *b* in the .swc file, the length of soma[*a*] in the .nrn file is defined using the x,y values of *a* and *b* and the diameter of soma[*a*] is calculated using the radius value of *a* from the .swc file. These values are calculated using the math Python library. The entire NRN-EZ session, which contains the stimulation parameters, and the cell morphology can be loaded by clicking the “Reload Session” button. Users can press the “Load .nrnez” button to upload a set of default parameters that they find convenient to have as part of their default screen. A sample .nrnez file is included in the NRN-EZ GitHub repository.

Once the .swc file is uploaded, one can visualize the reconstructed cell in the bottom portion of this panel. This plot relies on a custom implementation that uses the PyQtGraph Python graphics and GUI library built on PyQt/PySide and numpy. PyQtGraph was chosen over matplotlib after testing found that plotting with PyQtGraph was orders of magnitude quicker than using matplotlib (i.e., seconds vs. minutes). Using the definitions in the .swc file, the segments are plotted on a 2D graph. To reduce overhead further (i.e., time needed to display the plot), the plot is only written when a branching point is detected (with a branch being defined as a point with non-sequential parent id in the .swc file). Each segment type is given its own color (black for soma, blue for apical, cyan for basal and magenta for axon). The width of the line on the plots for the axon segment is also weighted 50% thinner than the other lines. PyQtGraph supports zooming in and out on the graph as well as panning. The axis units change from µm to mm depending on the size of the cell. The GUI displays the segment composition of the cell as a list that includes the number of segments in the soma, axon, apical and basal dendrites sections. In the interactive 2D Cartesian plane embedded in the GUI, these sections appear as color-coded in black (soma), magenta (axon), blue (apical dendrites) and cyan (basal dendrites). By right clicking and dragging the mouse up and down, we can zoom in and out the Cartesian reference system along the y-axis. By right clicking and dragging the mouse right and left, we can zoom in and out along the *x*-axis. The mouse scroll wheel can be used to zoom in simultaneously along the *x,y*-directions. By clicking and dragging the left mouse button, we can move the neurons around, in any direction. There is also a custom implementation to select a specific segment. The identity of a segment can be revealed in the fields above the graph by drawing a line across it. Briefly, by clicking once on either side of a given segment, the segment is highlighted with its complementary color, and its id is shown in the GUI. The selection is done by calculating a line based on the two points selected and then checking each segment to see if the segment intersects the calculated line. Users can also highlight specific segments by typing their segment identity number and segment identifier in the corresponding fields above the graph area (e.g., by typing “100” and selecting “Apical” from the dropdown box).

The left portion of the GUI also allows selecting the destination folder for the output files generated by NRN-EZ, which includes information about the weight, location and activation time of various synaptic inputs, or the amplitude and duration of current step injections. This can be done by navigating to the destination folder using the “Set Path” button, and then clicking the ”Run” button to execute the code specified through all other panels in the GUI. Users can select to run the code one or multiple times by typing a number in the field on the right-hand side of the “Run” button. To clear all parameters from the whole GUI, one can use the “Reset Fields” button on the right-hand side of the “Run” button.

### The middle panel of the GUI: selecting modules and setting their weight properties selection

In the middle panel of the GUI, users can select the type of stimulus (or module) to be used in their simulations. There are three main types of modules that can be used and that can be distributed across one or more segments. The “I-Step” module allows injecting current steps of any given amplitude and duration into a specified segment of a given compartment. The second module, called “Synaptic Input” is used to generate synaptic inputs based on a two-state kinetic scheme synapse described by rise time and decay time constants tau1 (rise) and tau2 (decay), respectively (see NEURON documentation). The name of each “I-Step” and “Synaptic Input” module type can be set arbitrarily by the user (e.g., AMPA, NMDA, step) in the box called “Name”, a useful feature to prevent potential nomenclature ambiguities when using multiple types of current steps or synaptic inputs in the same simulation. The third module type, “Custom”, allows the use of point processes (i.e., voltage or ligand-gated ion channels) with custom properties defined using .mod files. When using the “Custom” module type, the “.mod File” button prompts users to navigate to the .mod file to be used. In this case, once the .mod file is selected, NRN-EZ automatically sets the name the module in the table to match the name of the .mod file. Once the module type name has been assigned, users can select whether the weight of each input should remain the same (“Single”) or vary according to a uniform (“Multiple Uniform”) or Poisson distribution (“Multiple Poisson”). If the “Multiple Uniform” or “Multiple Poisson” options are selected, then the GUI prompts the user to select the mean and standard deviation of the current (“I-Step”) or of the synaptic weight (“Synaptic Input” and “Custom”). When using the “Synaptic Input” module type, the users also need to select the reversal potential (“E”), and the rise and decay time constants (tau1 and tau2, respectively). Once these parameters have been set, they are saved together with the module type and name and are displayed in the table at the center of this panel. The parameters of any saved module type can be changed by clicking on their corresponding row in the table, and they can be stored by clicking the “Save” button. The “Delete” button can be used to delete any current step or synaptic input that is no longer needed.

The bottom portion of the panel can be used to set the location and spatial distribution of each module type. The options for the spatial distribution are analogous to those mentioned when describing the synaptic weight. Accordingly, any given module type can be delivered to a single location identified by its segment number (“Single”) or to multiple locations according to either a uniform (“Multiple Uniform”) or Poisson distribution (“Multiple Poisson”). The mean of their spatial distribution is calculated using Euclidean distance from the user-selected segment.

### The right panel: stimulus tags, saving tags, and input timing

The right panel is used to label, or tag, a group of module types. Modules with the same tag are applied to the same exact locations and are activated at the same time. For example, assigning the same tag to both AMPA and NMDA module types can be used to create synapses that contain both AMPA and NMDA receptors at the same exact location. The user can select how many of these inputs should be applied to their cell of interest. The location of stimuli labelled by the same tag can be confined to one or more specific domains of the cell, for example the basal and/or apical dendrites. One can use a color-coded scheme for each tag. NRN-EZ will generate random distributions for stimulus magnitude, location and timing, independently for each stimulus tag. Users can decide the temporal pattern of activation of inputs with a given tag. These inputs can be delivered simultaneously by selecting “Single” and setting the variable “Interval” to zero. They can also be delivered with a constant time interval between each other (“Single”) or with a uniform (“Multiple Uniform”) or Poisson temporal distribution (“Multiple Poisson”), each defined by a mean time interval and standard deviation. The “Onset” field is used to set a delay between the beginning of the simulation and the activation time of the first input. “Interval” defines the time interval between consecutive stimuli. The table of saved tags in the right panel functions similarly to the saved stimuli table in the middle panel, where users can delete or edit the parameters assigned to a tag by selecting its corresponding row in the table.

### Generating the inputs and running the simulation

Once all the desired stimulus tags have been created, the code can be run by clicking the “Run” button in the bottom left corner of the GUI. After clicking the “Run” button, NRN-EZ displays each input in the Cartesian coordinate system of the left panel as a dot with a different color for each tag. The output files of NRN-EZ (.mod, .hoc, .py, .nrn, .pkl, .swc) are stored in a folder named with the date and time of its creation, in the destination folder identified by the “Set Path” button (left panel). The folder created by NRNR-EZ contains sub-folders corresponding to the number of runs the user selected. Within each run folder, there are .hoc and .py files containing commands to run the experiments in NEURON using either the hoc or Python interface, along with the .swc selected by the user, the .nrn file generated by the application, and all .mod files needed to run the simulation. NRN-EZ compiles the .mod files with the nrnivmodl command at the time of generation. Additionally, there are sub-folders corresponding to each module defined in the GUI. These folders contain multiple .dat files with input location, time, and weight information. In each .dat file, the first row contains two numbers, corresponding to the number of rows and columns in the file. All these files are used to run a Hodgkin-Huxley simulation using NEURON.

To run a NEURON simulation using the hoc interface, users can run the nrnez.hoc file, created by NRN-EZ and stored in its output folder. Alternatively, to run a NEURON simulation using the Python interface, users can run the nrnez.py file from the terminal with Python in interactive mode using the command “python nrnez.py -i”. Linux users that installed NEURON using the “pip” command in any location that required the use of the “sudo” command will also need to use “sudo” to run the .hoc or .py files. Each of these files contains an xopen command to open the .nrn file, which includes information about the cell morphology and mechanisms (i.e., the whole experiment) defined in NRN-EZ. Running the nrnez.hoc file creates a .ses (session) NEURON file containing all the parameters specified for the simulation, which can be changed from the NEURON GUI (e.g., simulation length). Included in the output files generated by NRN-EZ is the original .swc file, which one can use to take advantage of the NEURON Import3D function to check and correct for inaccuracies and potential discontinuities in the neuron morphology.

### Testing and validation

We tested and validated NRN-EZ by using the 3D morphology of a CA1 pyramidal cell (CA1-PC) in the mouse hippocampus, obtained using biocytin fills and confocal laser scanning microscopy image acquisition (NeuroMorpho NMO_139428)^47^. We assigned this neuron a resting membrane potential of − 65 mV, and the passive and active membrane properties of a CA1-PC described previously^47^. Briefly, the model included voltage-gated sodium channels (Na_V_), A-type potassium channels (K_A_), delayed-rectifier potassium channels (K_DR_), calcium-dependent potassium channels (K_Ca_), muscarinic receptor activated potassium channels (K_M_), hyperpolarization activated cationic channels (I_h_), and L, N, T-type calcium channels. All simulations were performed using a variable time step and the code for the NRN-EZ simulations is deposited on GitHub (https://github.com/scimemia/NRN-EZ). The NEURON code is shared on the ModelDB database (Acc n. 267419).

In a first series of simulations, we used NRN-EZ to change the location of a single excitatory input, with a conductance of 1 nS, from 100 µm (segment #3285) to 200 µm (segment #943) and 300 µm away from the soma (segment #2420; Fig. 3). Using the middle panel of the NRN-EZ GUI, we set the reversal potential for AMPA EPSPs at E = 0 mV. Each AMPA input had a rise time constant tau1 = 0.7 ms and a decay time constant tau2 = 2 ms. We selected “Single” location and entered the corresponding segment number and type (e.g., 3285 Apical) and pressed Save. In the right panel, we tagged this input as either Proximal, Medial or Distal and set the number of inputs to 1. The location limit was set to Apical Dendrite, to match the location of the selected segment. We selected red as the color for visualizing the input (Fig. 3A). At the bottom of this panel, for timing, we selected “Single”, set the Onset to 200 ms, clicked the Save button and then the Run button in the left panel. The results of the NEURON simulations performed using these files are shown in Fig. 3B. The amplitude of the somatic EPSP decreased as the input location moved further away from the soma, while its kinetics (rise and decay time) became slower. These findings are consistent with the basic principles of cable theory, used to describe the physiological properties of passive and active input propagation along dendrites^48^. If rather than using a single input, we used 100 of them, and distributed them with a Poisson location centered 100–300 µm away from the soma, we recorded a single action potential at the soma, arising on top of an EPSP that became less prominent for more distal inputs (Fig. 3C,D). When the excitatory inputs were not synchronous, but had a Poisson distribution for their activation time, the cell produced multiple action potentials if their location was < 200 µm away from the soma (Fig. 3E). A train of action potentials could be generated by varying the synaptic weight using a Poisson distribution (Fig. 3F). This is important, as it suggests that varying the strength and timing of the excitatory inputs increases the firing output probability of CA1-PCs, and therefore the temporal fidelity with which they relay information to the entorhinal cortex. The frequency of the trains decreased as the inputs became more distal (Fig. 3F). We detected a qualitatively similar trend when using a uniform rather than a Poisson distribution for synaptic input timing and weight. However, in this case, the firing output of the cell decreased more abruptly at increasing distances from the soma (Fig. 3G,H).

To understand how variability in the location, strength, and timing of activation of different synaptic inputs affect the integration of sub-threshold inputs, we repeated the simulations using a smaller conductance for the AMPA input (7.5 pS; Fig. 4). In this case, we quantified the maximal depolarization at the soma and the time integral of the change in membrane potential (i.e., the area under the curve, AUC). Consistent with the data previously described for Fig. 3A,B, a single excitatory input generated a smaller and slower EPSP as it moved from proximal to distal locations (Fig. 4A,B). This general principle held true also when analyzing the somatic output in response to 100 synchronous (Fig. 4C,D) and asynchronous inputs (Fig. 4E,F). The magnitude of the somatic depolarization, however, was larger when the value of the synaptic weight varied according to a Poisson distribution (Fig. 4F). This is consistent with the results described for supra-threshold stimuli (cf. Fig. 3E,F and 4E,F). This effect is less pronounced when the variability of synaptic weights is described through a uniform, rather than a Poisson, distribution (Fig. 4G,H).

The GABA_A_ component had a rise time constant tau1 = 0.5 ms and a decay time constant tau2 = 7 ms. The reversal for GABAergic synaptic inhibition was E = − 75 mV, meaning that its driving force was substantially smaller than that of glutamatergic excitation. Therefore, the general principle of distance-dependence for the attenuation of synaptic inputs held true but were smaller (though qualitatively similar) compared to those described for sub-threshold excitation. (cf. Figs. 4 and 5). Even in this case, the Poisson distribution of synaptic weights was more effective than the uniform distribution at hyperpolarizing the somatic membrane potential.

Together, these results confirm some fundamental principles of cable theory but also allow exploring new parameters related to the variability in the spatial and temporal properties of synaptic integration in central neurons.

## Discussion

Over the years, NEURON has become established in the neuroscience community as a powerful simulation environment capable of handling complex neuron geometry and biophysical mechanisms, essential to prompt and constrain new hypotheses on neuronal function. It has indirectly contributed to the establishment of major scientific enterprises, like the Human Brain Project, Active Brain Mapping and Human Connectome Project, which aim to find the connection between the cellular principles of neuronal coding and cognitive/motor performance^49–55^.

An important feature of NEURON, a simulation environment to run compartmental models of individual neurons and networks, is that it allows users to test how neuronal architecture, ion channel and synaptic input distribution and timing of activation affect the firing output and computational efficiency^56^ (see nrn.readthedocs.io/). Since its first development, NEURON has been used in more than 2600 publications focusing on topics that range from integration, basic mechanisms of synaptic transmission, dendritic spike initiation, to neuron excitability, etc. (see https://nrn.readthedocs.io/en/latest/publications-using-neuron.html). Although Python wrappers for NEURON have been developed, until 2022, NEURON incorporated a programming language based on hoc, which users could use for writing codes to define the weight and kinetics, spatial distribution, and activation time of synaptic inputs or the location of points for current injections^14^. In 2022, a Python interface was extensively modernized and gained some independence from hoc. However, having the ability to read hoc code written and shared by other groups, can still be a preferred entry point to learn NEURON for some users.

For many investigators with limited computer science knowledge, as well as undergraduate and graduate trainees that do not major in computer science but remain interested in understanding information processing in the brain, the learning barrier for NEURON and hoc can be high. This is particularly true when attempting to simulate complex activation patterns for different types of synapses with unique spatial distributions. In this work, we describe an application, NRN-EZ, which streamlines .hoc and .py file generation to run compartmental models, and provides the flexibility to run them using either the hoc or Python NEURON interface. The software source code and documentation for NRN-EZ are freely available for download from the Scimemi Lab website (https://sites.google.com/site/scimemilab2013/software) and GitHub repository (https://github.com/scimemia/NRN-EZ). Detailed instructions on software installation and operation are available from the GitHub repository, which also includes sample .swc files of 3D digitally reconstructed neuron morphologies. NRN-EZ can be downloaded as a standalone application for Linux (Pop!_OS and Ubuntu), Mac OS, and Windows. NRN-EZ is designed to facilitate the implementation of complex compartmental models by users with limited computer science experience. Being an open-source code, NRN-EZ can be implemented by anyone, which will be particularly useful for researchers around the world. NRN-EZ is flexible to cover a broad diversity of simulation scenarios, from current step injections to synaptic input activation. It allows to simulate synapses with mixed receptor populations, like glutamatergic synapses with AMPA and NMDA receptors.

We aim to keep NRN-EZ as simple, versatile, and useful as possible to make it a perfect entry tool to facilitate NEURON computations. Being an open-source tool will allow users around the world to contribute to future implementation, which can include, for example, the implementation of an interface to write hoc codes for neural network simulations.

## Materials and methods

### Software description

NRN-EZ is an open-source application that uses the Python programming language (version 3.6.9 and higher), the PyQt application (version 5.10.1) and the PyQtGraph module (version 0.11.0), built with PyInstaller 3.6. NRN-EZ was built on Linux (Ubuntu 18.04.5 LTS and Pop!_OS 18.04 LTS), MacOS Monterey 12.5.1 and Windows 10. NRN-EZ uses a Model—View—Controller (MVC) software design, which divides the program’s logic into three interconnected elements through which users can generate one or more synaptic inputs to run compartmental models using NEURON.

The Model portion of NRN-EZ consists of the following classes: experiment (also referred to as session in the GUI), morphology, weight, location (which also handles the number of inputs) and timing. The morphology class loads a .swc file, generates an output graph shown in the left panel of the GUI, and creates a .nrn file. The weight, location and timing classes all do error checking on the user input data and they all handle generating data based on the user input. The experiment class (i.e., the session) does error checking, and generates .hoc files. Any data with Poisson and uniform distributions are created using Python’s built-in math module. These data persist in the memory while the application is running. These and any other user-defined input parameters are also saved when the user hits the “Run” button in the bottom left corner of the GUI. When saving the data, NRN-EZ “pickles” the data (pickling is a built-in python module that saves python objects to flat files). Once saved, these .pkl files can be reloaded as a session into NRN-EZ at any time. The Model portion saves information into the following files: experimentClass.py, morphologyClass.py, locationClass.py, timeClass.py, weightClass.py. These files contain the class definitions for all the user input data. These also contain the output generation functions.

The View portion of NRN-EZ consists of the GUI built using PyQt. The plot section of the GUI is built using PyQtGraph, because of its ability to quickly generate large plots, like the one embedded in the NRN-EZ GUI. Accordingly, when users select a .swc file to load into NRN-EZ, the software quickly renders it graphically. The View portion renders information using the following files: gui.py, gui_helper.py. These files contain all the code for building the user interface.

The Controller portion of NRN-EZ transfers data from the View to the Model portion (e.g., when a user clicks the “Save” buttons in the middle and right portions of the GUI). In addition, the Controller portion transfers data from the Model to the View portion (e.g., when the user edits a module by clicking on its corresponding row in the tables or when the “Run” button is hit to execute the code and update the graph). The Controller portion handles data transfer using the gui_handler.py file. This file contains the code to handle user actions.

The code also includes a file that checks for command line arguments and is the entry point to the application (NRN-EZ.py), an error logger module (errorLogger.py), which generates the run log, error log, and debug log. It also handles generating error messages. The configuration module (config.py) handles options that users can change without having to recreate and version the code (i.e., the reference comment for the .hoc file outputs, setting a default .nrnez file and setting the debug mode on and off.). The configuration menu can be accessed by running NRN-EZ from the command line with the “-config” flag (i.e., “./NRN-EZ -config”). Additional command line flags include “-v” to display the version of NRN-EZ and “-d” to run the application in debug mode. Debug mode creates additional logs for troubleshooting the application. Loading .nrnez interface files is handled by the loader.py file. Last, the code stores information about the definitions of all global variables (globvar.py).

## Data Availability

The datasets generated and/or analyzed during the current study are available in the Scimemi Lab GitHub repository, https://github.com/scimemia/NRN-EZ.
